# Supplementary material for: Lipid Reshaping and Lipophagy Are Induced in a Modeled Ischemia-Reperfusion Injury of Blood Brain Barrier
Source: Int J Mol Sci. 2019 Jul 31;20(15):3752. doi: 10.3390/ijms20153752 (PMC6696511; doi:10.3390/ijms20153752)
Supplement: Supplementary file 1 [file ijms-20-03752-s001.pdf]

Table S1. Total fatty acid composition of RBE4 endothelial cells after OGD/ogR treatment for 1 and 24 h.

	µg/mg Prot						µg/10^6 Cells					
	CTRL		ogR1h		ogR24h		CTRL		ogR1h		ogR24h	
	mean	±S.E.	mean	±S.E.	mean	±S.E.	mean	±S.E.	mean	±S.E.	mean	±S.E.
C16:0	31.909	3.671	37.291	6.334	40.902	12.214	4.437	0.416	6.809	1.096	6.645	1.096
C16:1	11.511	1.197	13.035	1.697	12.076	4.566	1.603	0.135	2.380	0.290	1.919	0.459
C18:0	22.208	2.396	25.879	4.125	28.823	7.358	3.096	0.280	4.721	0.700	4.727	0.577
C18:1	85.942	9.884	99.709	12.892	109.430	31.817	12.044	1.301	18.223	2.272	17.761	2.688
C18:2	9.011	1.270	9.995	1.024	10.532	2.636	1.249	0.147	1.827	0.181	1.726	0.193
C18:3 n-3	0.913	0.146	0.942	0.196	1.204	0.497	0.124	0.013	0.171	0.032	0.190	0.052
C20:3	1.932	0.175	2.329	0.138	2.797	0.705	0.273	0.027	**0.425**	**0.022**	**0.458**	**0.052**
C20:4 n-6	10.122	0.662	**14.042**	**0.462**	15.110	2.005	1.450	0.169	**2.579**	**0.207**	**2.550**	**0.062**
C20:5	1.054	0.167	**1.893**	**0.235**	1.653	0.496	0.155	0.031	**0.343**	**0.029**	0.267	0.042
C22:5	4.279	0.297	5.934	0.704	6.761	1.822	0.601	0.041	**1.084**	**0.123**	1.103	0.144
C22:6	4.818	0.291	**7.046**	**0.366**	7.676	1.416	0.691	0.080	**1.292**	**0.100**	**1.279**	**0.071**
Total FA	183.698	19.415	218.095	27.379	236.964	65.476	25.722	2.511	39.855	4.809	38.625	5.363

Data are reported as mean ± S.E. from three independent experiments, bold numbers indicate a significant statistical difference vs. Control ($p < 0.05$).
